# Magnesium in Diabetes Mellitus and Diabetic Kidney Disease

**DOI:** 10.3390/jcm15197723

**Published:** 2026-10-07

**Authors:** Guido Gembillo, Luca Visconti, Luca Soraci, Lorenzo Lo Cicero, Manuel Scapellato, Concetto Sessa, Walter Morale, Antonino Catalano, Andrea Corsonello, Alberto La Spada, Ankit Sharma, Michele Provenzano, Domenico Santoro

**Affiliations:** 1 Unit of Nephrology and Dialysis, Department of Clinical and Experimental Medicine, University of Messina, 98125 Messina, Italy; guidogembillo@live.it (G.G.); manuelscapellato@gmail.com (M.S.); laspada.alberto@gmail.com (A.L.S.); dsantoro@unime.it (D.S.); 2 Unit of Nephrology and Dialysis, Ospedali Riuniti Villa Sofia Cervello, 90146 Palermo, Italy; a.corsonello@inrca.it; 3 Unit of Geriatric Medicine, Italian National Research Center on Aging (IRCCS INRCA), 87100 Cosenza, Italy; 4 PhD Program in Molecular and Clinical Medicine, University of Palermo, 90100 Palermo, Italy; lorenzolocicero95@gmail.com; 5 Department of Nephrology and Dialysis, Maggiore Nino Baglieri Hospital, 97015 Modica, Italy; concettosessa@gmail.com (C.S.); morale@asp.rg.it (W.M.); 6 Department of Clinical and Experimental Medicine, University Hospital of Messina, 98125 Messina, Italy; catalano.antonino@unime.it; 7 Department of Pharmacy, Health and Nutritional Sciences, University of Calabria, Rende, 87036 Cosenza, Italy; michele.provenzano@unical.it; 8 Department of Renal Medicine, University Hospitals of Coventry and Warwickshire, Coventry CV2 2DX, UK; ankit.sharma1@nhs.net

**Keywords:** magnesium, type 2 diabetes mellitus, diabetic kidney disease, TRPM6, insulin resistance, cardiovascular outcomes, SGLT2 inhibitors, hypomagnesemia

## Abstract

**Highlights:**

- How common is hypomagnesemia in diabetes? Hypomagnesemia has been reported in roughly one fifth to one third of people with type 2 diabetes, and its frequency appears to rise as diabetic kidney disease progresses.
- What drives renal magnesium loss? Reduced TRPM6 activity in the distal convoluted tubule has been linked to renal magnesium wasting and to insulin resistance, and the comparison between type 1 and type 2 diabetes fits this model.
- Does low serum magnesium predict end-stage kidney disease? In diabetic kidney disease, low serum magnesium was independently associated with progression to end-stage kidney disease, an association not observed in a parallel non-diabetic CKD subgroup.
- What does the magnesium depletion score show? Higher scores were associated with greater all-cause mortality in diabetic kidney disease. The score is a composite clinical risk index and does not measure body magnesium.
- Does oral magnesium lower cardiovascular events? Observational data associate oral repletion with lower cardiovascular event rates, an association confined to patients with documented hypomagnesemia.

**Abstract:**

Magnesium is a cofactor in cellular energy metabolism and insulin signaling, with recognized effects on vascular tone and cardiac electrophysiology. Hypomagnesemia affects roughly one-third of people with type 2 diabetes mellitus (T2DM) in pooled observational data and becomes more frequent in diabetic kidney disease (DKD). Reduced dietary intake accounts for part of the deficit; renal magnesium wasting linked to attenuated insulin signaling and reduced TRPM6 activity at the distal convoluted tubule is a plausible mechanism for the remainder, characterized mainly in cellular and experimental systems. In observational cohorts, lower serum magnesium accompanies poorer glycemic control and an excess of cardiovascular and microvascular events, and in DKD it independently predicts progression to kidney failure. Prescribed oral repletion has been associated with fewer cardiovascular events in hypomagnesemic patients, with no corresponding association in normomagnesemic ones, in propensity-matched data that cannot substitute for randomized confirmation. Mendelian randomization offers suggestive evidence for coronary artery disease and none for incident T2DM, a dissociation compatible with insulin resistance acting upstream of renal magnesium wasting, although instrument limitations leave other readings open. Hypomagnesemia is substantially less frequent in adult type 1 diabetes, where the association with glycemic control concentrates among patients with the highest insulin requirements. SGLT2 inhibitors raise serum magnesium modestly, whereas thiazide and loop diuretics and proton-pump inhibitors lower it. Randomized trials have shown modest effects on glycemic intermediates, larger in patients with documented deficiency, and no trial has yet tested renal progression. Magnesium status is a reproducible prognostic marker in T2DM and DKD; its value as a therapeutic target awaits randomized trials with clinical endpoints.

## 1. Introduction

The International Diabetes Federation estimates that in 2024, 589 million adults aged 20 to 79 years were living with diabetes, 11.1% of the world population in this age group, with 853 million projected by 2050, and type 2 diabetes mellitus (T2DM) accounting for roughly nine in ten cases [1]. Diabetic kidney disease (DKD), now the leading cause of end-stage kidney disease (ESKD) worldwide, accounts for a large share of the cardiorenal mortality increment in these populations [2,3,4]. Magnesium has re-emerged in cardiorenal research as a potentially modifiable contributor to glycemic control and to vascular and renal physiology. The deficit has two main sources: reduced dietary intake and renal wasting at the distal convoluted tubule, where attenuated insulin signaling reduces TRPM6 activity [5,6]. Hypomagnesemia is common in T2DM, and recipients of corrective therapy are a minority of those biochemically eligible in outpatient practice [7]. Cohort-level dose–response resolution in non-dialysis T2DM has been substantially advanced by the 1,284,940-patient veterans analysis of Yin and colleagues [7], which traced a parabolic serum-magnesium–major adverse cardiovascular events (MACE) relationship across the full reference range.

Against this background, DKD cohorts reproduce a progression signal that a parallel non-diabetic CKD subgroup largely fails to show, while genetic instruments for serum magnesium shift coronary artery disease risk, yet leave T2DM risk essentially unchanged. These patterns motivate the present synthesis of cohort epidemiology, randomized trial data, Mendelian randomization and biomarker analyses, which addresses one interpretive question: whether the magnesium–diabetes link is a feature of diabetes as a disease category or of the insulin-resistance physiology that dominates T2DM.

### Methodological Note

PubMed/MEDLINE, Embase and the Cochrane Central Register of Controlled Trials were searched from inception to August 2026 for English-language publications combining terms for magnesium biology (“magnesium”, “hypomagnesemia”, “TRPM6”, “magnesium depletion score”) with terms for diabetes phenotypes (type 1, type 2 and gestational diabetes, post-transplant diabetes, diabetic kidney disease), pharmacological exposures and cardiovascular and renal outcomes; the reference lists of retrieved articles, current KDIGO and ADA guidance and key reviews were screened by hand. Eligible records were original human studies (prospective and retrospective cohorts, registries, target trial emulations, randomized trials and their meta-analyses, Mendelian randomization analyses and biomarker studies) and mechanistic studies in cellular or animal systems informing renal magnesium handling; case reports were retained only where they illustrated a mechanism documented nowhere else, and conference abstracts, editorials and non-English publications were excluded.

Large prospective cohorts, randomized trials, meta-analyses and Mendelian randomization studies were given priority; smaller studies were retained where they provided mechanistic or comparator information unavailable in larger datasets. Synthesis is thematic given the heterogeneity of designs, assays and exposure definitions, and where the text states that a given type of evidence appears to be lacking, the statement refers to the searches described above at the date of resubmission.

## 2. Physiology and Renal Handling of Magnesium

The adult body contains approximately 22–26 g of magnesium. Around 60% sits in bone, 20% in skeletal muscle, 19% in other soft tissues and only 1% in the extracellular compartment. The serum pool is partitioned into an ionized fraction (~62%), a protein-bound fraction (~33%) and a small complexed fraction (~5%) [6,8]. The reference interval is typically 0.75–0.95 mmol/L (1.8–2.3 mg/dL), with cut-offs that drift across laboratories and guidelines [6,8]. Intestinal absorption operates through two routes: a passive paracellular pathway throughout the small bowel, and an active transcellular pathway mediated by TRPM6 and TRPM7 channels, concentrated in the distal colon [6]. The kidneys govern overall balance, and approximately 10–20% of the filtered load is reabsorbed in the proximal tubule and 60–70% paracellularly in the thick ascending limb, driven through claudin-16/19 tight junctions by a lumen-positive transepithelial gradient that NKCC2 activity and apical K^+^ recycling via ROMK together generate. Fine regulation belongs to the distal convoluted tubule, where TRPM6 mediates active transcellular reabsorption against the electrochemical gradient and integrates hormonal and metabolic signals, including insulin, that determine final urinary excretion [5,6].

When insulin engages its tubular receptor, a phosphoinositide 3-kinase signal recruits Rac1 and increases TRPM6 activity and surface expression at the apical membrane [5,9]. Attenuation of that signal reverses the sequence: channel abundance declines, urinary magnesium losses rise and serum magnesium drifts downward. This mechanism has been characterized in cell systems, and the Atherosclerosis Risk in Communities Study extended the observation to population scale, showing a significant interaction between a TRPM6 variant and circulating insulin on serum magnesium in European-American participants [10], compatible with the insulin-dependent regulation of TRPM6 previously characterized in vitro. Renal handling is therefore a plausible contributor to hypomagnesemia in T2DM alongside dietary intake, and inappropriate urinary magnesium losses have been documented in T2DM patients [11]; the relative weight of the two sources has not been quantified and probably varies with patient context.

## 3. Dietary Magnesium Intake and Incident Type 2 Diabetes

Across cohort data from North America, Europe and Asia, dietary magnesium intake has shown a consistent inverse association with incident T2DM. The cohorts summarized in this section enrolled general populations free of diabetes at baseline, so their estimates concern incident disease and cannot be transferred to outcomes in established T2DM. In the Framingham Offspring Study, participants in the upper range of magnesium intake had better insulin sensitivity and slower progression from prediabetes to T2DM across roughly seven years of follow-up [12]. Pooled cohort estimates settled around an inverse relative risk of 0.85 to 0.86 per 100 mg/day, with overlapping confidence intervals across the Larsson–Wolk seven-cohort and Dong thirteen-cohort syntheses [13,14]. Dietary magnesium is rarely consumed in isolation, as it naturally co-occurs with cereal fiber, polyphenols, and broader plant-rich eating patterns. Mendelian randomization offers one route to separating the mineral from its dietary context and is considered in Section 8. The Fang synthesis disaggregates by endpoint: across forty cohorts and over a million participants followed for between four and thirty years, per-100 mg/day relative risk was 0.81 (95% CI 0.77–0.86) for T2DM and 0.78 (0.69–0.89) for heart failure, weakening to 0.93 (0.89–0.97) for stroke and 0.90 (0.81–0.99) for all-cause mortality, and lying within sampling distance of unity for coronary heart disease (0.92, 0.85–1.01) and aggregate cardiovascular disease (0.99, 0.88–1.10) [15]. Magnesium intake consequently correlates with metabolic and cardiac-substrate endpoints, while the coronary and composite cardiovascular estimates lie close to unity, and observational dietary data alone do not explain the difference. Zhao and colleagues, in an updated meta-analysis through March 2019 [16], reported a concordant T2DM signal (RR 0.78, 95% CI 0.75–0.81 highest versus lowest intake) and an inverse association with total and ischemic stroke but not hemorrhagic; the highest-versus-lowest contrast in Zhao is not directly comparable with the per-100 mg/day estimates in Fang, although direction and broad magnitude agree across syntheses. Sensitivity analyses in Dong and colleagues restricted to studies adjusted for cereal fiber intake yielded results similar to the primary analysis [14], partially but not fully resolving the wholegrain confounder. The stroke signal weakens in East Asian cohorts relative to European and North American ones, plausibly reflecting differences in background dietary composition and, in particular, the calcium-to-magnesium intake ratio.

Larsson, Orsini and Wolk provided independent meta-analytic confirmation of the stroke association. Their meta-analysis of 241,378 participants, pooled across seven independent prospective cohorts, showed that every 100 mg/day increment in dietary magnesium was associated with a relative stroke risk of 0.92 (95% CI 0.88–0.97), with a stronger effect for ischemic than for hemorrhagic stroke [17]. None of these cohort syntheses can fully separate the magnesium signal from the broader dietary pattern in which it is embedded, or convert correlation into causation.

The serum-side evidence points in a concordant direction while carrying a different mechanistic implication. In 12,128 nondiabetic participants in the Atherosclerosis Risk in Communities study, with a follow-up of six years, low serum magnesium was a strong independent predictor of incident T2DM among white participants, whereas low dietary magnesium conferred no comparable risk, a dissociation the authors attributed to the compartmentalization and renal handling of magnesium, with intake accounting for only part of the circulating concentration [18].

## 4. Serum Magnesium in Type 1 Diabetes Mellitus

Adult type 1 diabetes mellitus (T1DM) combines chronic hyperglycemia with a generally lower burden of insulin resistance than T2DM, and therefore dissociates hyperglycemia from insulin-resistance physiology, allowing their separate contributions to renal magnesium handling to be examined. The most detailed characterization comes from the cross-sectional series of Oost and colleagues at Radboud University Medical Center. Of 241 adult T1DM patients, 2.9% met the <0.7 mmol/L hypomagnesemia threshold, roughly tenfold below T2DM cohorts of matched disease duration [19]. Across the whole cohort, magnesium and HbA1c correlated only weakly (r = −0.12, *p* = 0.068), with neither inflammatory markers nor adipokines tracking with serum magnesium. Stratification by daily insulin dose (IU/kg, entered as proxy for insulin resistance) altered this picture. In hierarchical regression adjusted for age, sex, diabetes duration, renal function, blood pressure, smoking and alcohol use, insulin dose moderated the magnesium–HbA1c association (B = −71.9 mmol/mol per mmol/L per IU/kg, 95% CI −119.1 to −24.7, *p* = 0.003; the magnesium–HbA1c slope steepens linearly across higher insulin-dose strata) and the magnesium–log_10_ high-sensitivity C-reactive protein (hsCRP) association (B = −2.09, −3.70 to −0.48, *p* = 0.011), with the inverse coefficients steepening progressively across the upper insulin-dose strata. Hypomagnesemia in adult T1DM concentrates in the subgroup phenotypically convergent with T2DM, with insulin requirement serving as an imperfect proxy for measured insulin sensitivity.

A second Dutch series of 207 adult T1DM outpatients (median diabetes duration 22 years and mean HbA1c 7.6%) returned hypomagnesemia at the same threshold in 4.3%, with mean serum magnesium 0.66 ± 0.03 mmol/L in the deficient subgroup [20]. Magnesium correlated with neither baseline HbA1c (r = −0.014, *p* = 0.843) nor with micro- or macrovascular complications, and where present was clinically mild. The Radboud and van Dijk prevalences (2.9% and 4.3%) are substantially below those reported for T2DM at comparable disease duration in available adult series; assay-, threshold-, age- and medication-related differences between cohorts limit precise quantitative comparison.

The pediatric pattern diverges: of 71 Egyptian children with T1DM (mean age 9.68 ± 3.99 years), 28.2% met the <1.7 mg/dL threshold, with diabetes duration the strongest independent predictor in regression analysis (*p* < 0.001) [21]. Open-label administration of magnesium oxide at 300 mg/day for three months in the deficient subgroup, without a parallel control group, raised mean serum magnesium from 1.45 ± 0.15 to 1.94 ± 0.12 mg/dL and lowered HbA1c from 10.11% to 7.88% (*p* < 0.001), with concurrent improvement in lipid profile. This HbA1c reduction is larger than those reported in adult randomized magnesium trials, and the single-center uncontrolled design does not allow it to be separated from concurrent intensification of routine diabetes care. A comparable inverse magnesium–HbA1c correlation has been reported in Greek pediatric T1DM [22], and a systematic review of seven T1DM studies addressing glycemic control identified the inverse association in five [23]. A detectable magnesium signal in T1DM emerges most clearly under rapid growth, metabolic instability and high insulin requirements, and is substantially attenuated in stable, middle-aged adult T1DM, persisting primarily in the high-insulin-dose subgroup [23].

These observations are difficult to reconcile with hyperglycemia-driven osmotic diuresis as the sole explanation, since the magnesium–HbA1c association is weaker in adult T1DM than in T2DM. The comparison across T1DM and T2DM series is nonetheless observational and does not isolate hyperglycemia from insulin resistance, because age, medication exposure, renal function, disease duration and assay methods all differ between these cohorts. The concentration of the magnesium–HbA1c association in the high-insulin-dose subgroup of adult T1DM [19] is compatible with insulin-resistance physiology contributing to renal magnesium handling alongside hyperglycemia. Daily insulin dose is an imperfect proxy for measured insulin sensitivity; the T1DM evidence rests on a small number of cross-sectional cohorts, and the comparison therefore generates a hypothesis that a study measuring insulin sensitivity and magnesium handling in the same patients would be needed to test.

## 5. Hypomagnesemia in Type 2 Diabetes Mellitus: Prevalence, Determinants and Pathophysiology

Hypomagnesemia prevalence in T2DM varies widely across published series, from 13.5% to 47.7% in the review by Pham and colleagues [24], although more recent estimates in well-defined primary-care cohorts are considerably lower: 9.6% in the 929-patient Dutch primary-care series of Waanders and colleagues [25]. A 2024 meta-analysis of 19 observational studies (4192 patients) placed the pooled prevalence at 32% (95% CI 22–36%) [26]. However, these figures carry a measurement caveat inherent to the assay used throughout this literature. Total serum magnesium is an imperfect proxy for whole-body magnesium status [8], and the true prevalence of magnesium deficiency in T2DM may be underestimated by serum-based assessment.

Prevalence figures are also sensitive to the threshold applied. Published series define hypomagnesemia variously as serum magnesium below 0.70 mmol/L, below 0.75 mmol/L, below 1.7 mg/dL or below 1.8 mg/dL, and the assays behind these cut-offs differ in calibration [6,8]. Because the distribution of serum magnesium in T2DM is centered close to the lower reference limit, a shift of 0.05 mmol/L in the threshold moves a substantial share of a cohort across the diagnostic boundary, so prevalence estimates and effect sizes obtained under different definitions cannot be compared directly. The figures quoted in this review are reported with the threshold used in each source, and differences between them should be read with this caveat in mind.

Gommers and colleagues framed the underlying architecture as a vicious circle [5]. At the distal convoluted tubule, insulin recruits TRPM6 to the apical membrane via a PI3K–Rac1 pathway that phosphorylates residues T1391 and S1583 [9]; under conditions of attenuated insulin signaling, channel surface expression has been shown to fall in cellular systems, and in T2DM patients, urinary magnesium losses rise consistently with this prediction [11]. The resulting intracellular deficit may in turn impair insulin receptor tyrosine kinase activity and GLUT4 trafficking and reduce glucose-stimulated insulin release from the beta cell, although these downstream steps rest predominantly on in vitro and animal evidence with limited direct human tubular confirmation. Mechanistic support comes from Nair and colleagues, who showed that the TRPM6 variants V1393I and K1584E lose insulin responsiveness in patch-clamp and TIRF assays and associate with higher glycated hemoglobin and gestational diabetes in a cohort of 997 pregnant women [9]. At population scale, the ARIC candidate-region analysis of Tin and colleagues reported a gene–insulin interaction at the TRPM6 index variant rs11144134 in European-American participants, with a 1.6-fold stronger association between rs11144134 and serum magnesium at lower fasting insulin levels (*p* for interaction = 0.03) [10]. The reciprocal arm of the proposed relationship draws on the fractional-excretion data of Kurstjens and colleagues [11], which documented inappropriate renal magnesium losses in T2DM patients independently of filtered load. The framework predicts that correction of the deficit should improve insulin sensitivity where magnesium is biologically limiting, and the randomized evidence bearing on this prediction is mixed: early trials in hypomagnesemic patients reported improvements in HOMA-IR and beta-cell compensation [27,28], whereas a double-blind crossover trial of 14 insulin-treated T2DM patients selected for serum magnesium at or below 0.79 mmol/L found that six weeks of oral magnesium gluconate raised serum magnesium without changing insulin sensitivity measured by hyperinsulinemic-euglycemic clamp [29] (Section 11). The bidirectional model therefore remains a hypothesis supported by cellular, animal and observational data, with direct human tubular confirmation and consistent interventional support both lacking.

Population-scale evidence is consistent with this architecture: within the Rotterdam Study, a general-population cohort of 8555 participants, serum magnesium predicted incident prediabetes and diabetes, and a formal mediation analysis attributed part of the association to insulin resistance quantified by HOMA-IR, while a parallel analysis traced an effect of common variation in magnesium-regulating genes acting through serum magnesium [30].

## 6. Macrovascular Outcomes in Type 2 Diabetes Mellitus

Magnesium relaxes vascular smooth muscle and antagonizes calcium at the ischemic myocardium in experimental systems, with additional effects on platelet aggregation and vascular inflammation [6,31]. The Dutch Diabetes Care System analysis by Oost and colleagues, published in 2021, provided the first adequately powered prospective T2DM-restricted analysis to test these mechanisms at the cohort level [32]. Across 4348 participants with mean baseline serum magnesium of 0.80 ± 0.08 mmol/L, macrovascular follow-up over 6.1 years returned hazard ratios of 0.87 (95% CI 0.76–1.00) for MACE (the DCS composite, broadly defined to include heart failure and atrial fibrillation alongside acute myocardial infarction, coronary heart disease, cerebrovascular accident and peripheral arterial disease), 0.76 (0.62–0.93) for heart failure and 0.59 (0.49–0.72) for atrial fibrillation, with no individual association for acute myocardial infarction, coronary heart disease, cerebrovascular accident or peripheral arterial disease. The same cohort’s microvascular follow-up over 5.1 years extended the inverse pattern to CKD (HR 0.89, 95% CI 0.82–0.96), diabetic retinopathy (0.77, 0.61–0.98), foot complications (0.85, 0.78–0.92) and the composite microvascular endpoint (0.85, 0.78–0.91). HbA1c mediated the heart failure, retinopathy, foot and microvascular-composite signals; the atrial fibrillation effect resisted glycemic-control mediation, locating its operative pathway outside glycemic control.

Cohort-level dose–response shape emerged at scale in the Yin Veterans Health Administration analysis [7]. Among 1,284,940 T2DM patients with at least one outpatient serum magnesium measurement between 1999 and 2021, 17.8% fell below 1.8 mg/dL and 9.2% exceeded 2.3 mg/dL. Against normomagnesemia (1.8–2.3 mg/dL) as referent, MACE hazard rose at both tails, by 11–20% across the lower and by 4–39% across the upper, with the parabolic structure carrying its nadir near the middle of the reference interval. Treatment uptake by prescribed oral magnesium reached only 9.7% of hypomagnesemic patients, against 0.7% of normomagnesemic ones, despite routine assay availability. Propensity-score matching across 64 baseline covariates produced, among 40,766 matched hypomagnesemic patients (20,383 pairs), a MACE hazard ratio of 0.89 (95% CI 0.84–0.93) under prescribed repletion, with the benefit driven by patients concurrently exposed to proton-pump inhibitors or thiazide diuretics; the parallel analysis in 11,838 matched normomagnesemic patients (5919 pairs) returned a null result (HR 1.07, 0.97–1.17). These findings raise the hypothesis of an optimal serum magnesium window in T2DM. The parabola describes the distribution of risk across observed concentrations in one cohort; it does not identify a concentration at which intervention changes outcome, no trial has treated to a target within the reference range, and the nadir of the curve should not be read as a supplementation target. Any cardiovascular benefit in these data is confined to patients with a documented deficit. Generalizability is the principal limit, since the Veterans Health Administration cohort over-represents older US-based men with heavy cardiovascular and renal comorbidity, narrowing the population to which the parabolic curve can be extrapolated. Propensity-score matching, however well-balanced across measured covariates, is not randomization; residual confounding by indication, adherence and unmeasured comorbidity remains. No dataset of comparable size appears to have been published, and the review is consequently weighted toward this single source for adjudicated cardiovascular outcomes. Independent replication in demographically distinct cohorts and, ultimately, a randomized trial of repletion in hypomagnesemic T2DM are needed.

A related target trial emulation by Cheng and colleagues, drawn from the same VA research group, assembled 94,239 veterans aged 40 or older with diabetes and no prior heart failure or magnesium use; natural language processing of clinic notes flagged 17,619 self-reported users of nonprescription magnesium supplements (mean duration of use 3.5 ± 3.1 years) against 76,620 nonusers [33]. Inverse-probability-of-treatment weighting across 88 covariates produced incident heart failure rates of 8.0% in users and 9.7% in nonusers (HR 0.94, 95% CI 0.89–0.99); the composite of myocardial infarction, stroke, heart failure hospitalization or death moved by the same coefficient (HR 0.94, 0.90–0.97). The absolute risk reduction is small, and confounding by indication and the limits of natural-language-processing exposure ascertainment cannot be set aside; the coefficient carries information about the direction of the association and leaves its precise magnitude uncertain. Its agreement in direction with the Oost and Yin signals, across designs that share little beyond the magnesium exposure, adds convergent support from an independent design.

Earlier evidence points the same way: in the Framingham Offspring analysis, Hruby and colleagues found dietary magnesium intake inversely associated with coronary artery calcification [34]. Within the Atherosclerosis Risk in Communities cohort, Liao and colleagues documented an inverse association of serum magnesium with incident coronary heart disease [35] and Ohira and colleagues documented a comparable inverse relationship with ischemic stroke [36]. de Baaij and colleagues lay out the mechanistic picture in their Physiological Reviews synthesis, covering magnesium in arrhythmogenesis, endothelial function and vascular tone [6]. The heart failure signal is corroborated on the dietary side by Fang and colleagues [15]: a pooled RR of 0.78 per 100 mg/day increment of similar magnitude to the serum magnesium HR of 0.76 for incident heart failure in the Oost DCS cohort [32].

## 7. Microvascular Complications: Retinopathy, Neuropathy and Diabetic Foot

Microvascular complications in T2DM share an endothelial dysfunction substrate in which oxidative stress and microthrombotic activation operate against a background of cumulative advanced glycation end-product injury [2,37]; magnesium deficiency intersects this substrate at multiple points [37].

Across published series, lower serum magnesium tracks consistently with the presence and severity of diabetic retinopathy. The earliest, reported by Hatwal and colleagues in 1989, stratified 100 Indian T2DM patients by retinopathy grade against 100 matched controls [38], with the methodological caveats of pre-modern total-serum-magnesium assays. In patients with malnutrition, hepatic disease, renal disease or albuminuria excluded, serum magnesium was significantly lower in diabetic than in non-diabetic controls (*p* < 0.001) and lower again across retinopathy strata (*p* < 0.001). The North Indian series of 250 T2DM patients by Kumar and colleagues, in which 44% were hypomagnesemic at the ≤1.7 mg/dL threshold, reproduced the pattern across the glycemic axis (worse fasting and post-prandial glycemia and HbA1c in the hypomagnesemic patients) and across retinopathy stage (non-proliferative retinopathy at 62.7% and proliferative at 21.8% in hypomagnesemic patients) (*p* < 0.001) [39]. Retinopathy prevalence climbed monotonically across descending serum magnesium quartiles in the 2222-patient T2DM series of Xing and colleagues, from 21.0% at Q4 (≥0.96 mmol/L) through 23.5% and 30.2% to 50.9% at Q1 (≤0.85 mmol/L), yielding an adjusted OR of 3.91 for Q1 versus Q4 and an ROC cut-off of 0.875 mmol/L (2.13 mg/dL) for retinopathy prediction [40]. The same vector emerged in 104 T2DM patients with preserved renal function classified by Early Treatment Diabetic Retinopathy Study criteria, where mean serum magnesium fell stepwise, from 1.76 ± 0.22 mg/dL in patients without retinopathy through 1.63 ± 0.30 mg/dL with retinopathy (*p* = 0.029) to 1.55 ± 0.33 mg/dL in sight-threatening disease (ANOVA *p* = 0.031); the renal dysfunction exclusion isolated the retinopathy signal from nephropathy-related confounding [41]. Application of the magnesium depletion score to NHANES 2005–2018 data confirmed the association with retinopathy in T2DM [42].

Magnesium shows a weaker and less consistent relationship with neuropathy. In a cohort of 978 T2DM patients with formal nerve conduction studies, serum magnesium was lower in those with abnormal conduction than in those without (median 0.87 vs. 0.88 mmol/L, *p* = 0.048), with composite amplitude z-score rising stepwise across magnesium tertiles, from −0.60 in the lowest to −0.48 in the highest (*p* for trend = 0.001) [43]. The 254-patient Huashan Hospital series replicated the association, with one analytically relevant detail: phosphate moved with magnesium, both ions lower in neuropathy cases (*p* < 0.01 each) and both positively associated with amplitude and conduction-velocity z-scores in models adjusted for age, sex, smoking, disease duration, body mass index, systolic blood pressure, HbA1c, total cholesterol and estimated glomerular filtration rate [44]. The parallel reduction in two divalent cations is in keeping with a shared tubular handling defect, not an isolated magnesium effect. Strom and colleagues extended the analysis to mechanism in 235 newly diagnosed T2DM patients from the German Diabetes Study baseline cohort, confirming lower serum magnesium in the 51 patients with sensorimotor polyneuropathy compared with the 184 without and, against expectation, finding no association of plasma methylglyoxal concentration with polyneuropathy on its own [45]. In SH-SY5Y cells and in mouse dorsal-root-ganglion preparations, the same group showed that magnesium was protective against methylglyoxal-induced toxicity, with magnesium supplementation preventing neurite degeneration linked upstream to mitochondrial downregulation and triose-phosphate accumulation. These experimental data position the magnesium deficit as a determinant of how much AGE-related injury a given neuron can tolerate, a reading that rests on cell and animal systems and awaits confirmation in human nerve.

Farvid and colleagues conducted a randomized placebo-controlled trial in 75 T2DM patients with peripheral neuropathy: oral supplementation with a micronutrient containing magnesium (250 mg/day) improved several symptoms of diabetic neuropathy on the MNSI questionnaire after four months in both groups treated with micronutrients and magnesium supplementation, although the specific contribution of magnesium could not be distinguished from that of the other nutrients administered [46].

Diabetic foot complications arise at the convergence of neuropathy and microvascular disease. Rodríguez-Morán and Guerrero-Romero matched 33 T2DM patients with foot ulcers of less than two months’ duration against 66 T2DM controls of comparable age, disease duration, HbA1c and fasting glycemia, and found serum magnesium significantly lower in the ulcer group, with serum magnesium depletion present in 93.9% of cases versus 73.1% of controls and a logistic-regression odds ratio of 2.9 (95% CI 1.7–6.8) [47]. A single randomized placebo-controlled trial has addressed supplementation in this setting: Razzaghi and colleagues gave magnesium oxide 250 mg daily or placebo for 12 weeks to 70 patients with grade 3 diabetic foot ulcer and reported greater reductions in ulcer size together with improvements in glucose metabolism and inflammatory markers [48]. The result rests on a single small trial with a surrogate endpoint, and no trial with a healing or amputation endpoint appears to have been published.

## 8. Mendelian Randomization

The literature summarized so far is vulnerable to a specific form of reverse causation in which insulin resistance may itself drive renal magnesium wasting, so the observed inverse correlation between serum magnesium and diabetic outcomes could run in either direction, or in both. Mendelian randomization (MR) is the standard inferential tool for this configuration. Germline variants are allocated at meiosis, independently of lifestyle and later disease, so a variant that shifts serum magnesium up or down across a lifetime approximates random treatment assignment in a setting where the equivalent randomized trial is not feasible. The genetic material for magnesium MR derives from the Meyer 2010 CHARGE GWAS [49], which identified six genome-wide significant loci (in or near MUC1, ATP2B1, DCDC5, TRPM6, SHROOM3 and MDS1); these six SNPs have served as the instrument set for nearly all subsequent magnesium MR analyses.

A GWAS restricted to people with T2DM has since become available and bears on the transferability of these instruments. Oost and colleagues analyzed 3466 participants of European descent from the Hoorn Diabetes Care System cohort, in whom mean serum magnesium was 0.80 ± 0.08 mmol/L and 9.5% had concentrations below 0.7 mmol/L, with models adjusted for age, sex and principal components of ancestry and then additionally for eGFR and HbA1c [50]. A single locus in TAF3 (rs7894336) reached genome-wide significance (*p* = 2.9 × 10^−9^) and remained significant under eGFR and HbA1c adjustment; variants in the same region were linked in skeletal-muscle expression data to ATP5F1C, which encodes a subunit of mitochondrial ATP synthase, the complex that forms magnesium-ATP. MUC1/TRIM46, SHROOM3 and SLC22A7 reached nominal significance only, although the first two had already been identified in general-population GWAS and gave effect estimates in the same direction when meta-analyzed with the Meyer data. The TRPM6 signal of the general-population studies was not replicated, which the investigators attributed to a cohort composed largely of insulin-resistant individuals in whom insulin-dependent regulation of the channel may be impaired; the sample was too small to support Mendelian randomization against diabetic complications, and the investigators listed its size among the limitations of this study. Described by its authors as the first GWAS of serum magnesium conducted in T2DM, this study suggests that instruments derived from the general population may carry different weights, at TRPM6 in particular, in an insulin-resistant population.

Larsson, Burgess and Michaëlsson tested whether genetically predicted serum magnesium is causally associated with coronary artery disease in a two-sample design [51]. Their instrument pooled the six Meyer SNPs against CARDIoGRAMplusC4D summary data on 60,801 coronary artery disease cases and 123,504 non-cases drawn from 48 studies. The six-SNP instrument explained 1.62% of serum magnesium variance with a mean F-statistic of 64, above the conventional weak-instrument threshold of 10, although the absolute fraction of magnesium variance captured remains modest. Per 0.1 mmol/L (approximately one standard deviation) genetically predicted increment in serum magnesium, the odds ratio for coronary artery disease was 0.88 (95% CI 0.78–0.99, *p* = 0.03) under fixed-effects IVW, widening to 0.88 (0.74–1.05, *p* = 0.14) once between-instrument heterogeneity was admitted under random effects (P_heterogeneity = 0.06). Pleiotropy-robust sensitivity analyses moved in the same direction, with weighted median 0.84 (0.72–0.98) and heterogeneity-penalized model averaging 0.83 (0.71–0.96). The heterogeneity localized on leave-one-out to rs11144134 at TRPM6; excluding this variant collapsed P_heterogeneity from 0.06 to 0.74 and tightened the causal estimate to OR 0.82 (0.72–0.93, *p* = 0.002) under both specifications. TRPM6 loss-of-function variants produce hypomagnesemia with secondary hypocalcemia, and serum calcium has its own causal link to coronary disease; rs11144134 plausibly violates the exclusion-restriction assumption by acting through calcium as well as, or instead of, magnesium.

For T2DM, the MR signal is null: in a multi-trait MR by Cheng and colleagues, examining five common minerals against nine chronic-disease outcomes under IVW and weighted-median estimators, genetically predicted serum magnesium showed no significant causal effect on T2DM risk [52]. Cardiovascular endpoints were not in their outcome panel, so the analysis informs the diabetes question without addressing CAD, heart failure or atrial fibrillation. In the literature reviewed here, CAD signal remains the principal positive MR finding for serum magnesium in the cardiometabolic domain [51].

The cohort–MR discrepancy in T2DM tolerates more than one reading. Under a reverse-causation interpretation, with insulin resistance acting upstream of renal magnesium wasting (the Radboud stratification and the ARIC TRPM6×insulin interaction are compatible with this account), low magnesium would be a partly downstream marker of insulin-resistance physiology, with the driving lesion sitting above it in the causal sequence; a genetic instrument shifting lifetime serum magnesium by 0.1 mmol/L would not displace the driving lesion, and a null MR for T2DM would follow as prediction.

This dissociation should not, however, be read as directional proof of pathophysiology. The T2DM MR datasets are underpowered given the small fraction of magnesium variance that current instruments explain, residual pleiotropy at the six Meyer SNPs cannot be excluded, the Hoorn GWAS indicates that instrument weights derived from the general population may not apply unchanged to insulin-resistant patients [50], and binary disease endpoints are insensitive to the modest lifetime exposure shifts genetic instruments produce. A positive coronary signal can in any case persist independently of the T2DM null, since coronary atherogenesis admits pathways such as vascular tone, endothelial function, arrhythmia threshold and arterial calcification on which circulating magnesium may act without obligatory intermediation by insulin resistance. Larger MR studies with stronger instruments and mechanistic work in human tissue would be required to distinguish these accounts.

In 1323 pregnant women from the Tongji Maternal and Child Health Cohort genotyped by Cao and colleagues, combined mutant-allele burden across three magnesium-handling polymorphisms (two in TRPM6 and one in TRPM7) predicted gestational diabetes, with adjusted RR of 1.65 (95% CI 1.21–2.23) for women in the higher mutant-allele burden stratum versus those in the lower [53]. Cao and colleagues report a gene–environment interaction analysis, which falls short of formal MR while preserving much of its inferential logic: germline variants in magnesium-handling genes act as lifelong exposures, and their interaction with dietary intake addresses whether magnesium status sits upstream or downstream of glucose tolerance. Dietary magnesium below 370 mg/day was associated with lower plasma magnesium (15.34 vs. 15.80 mg/L, *p* < 0.001) and with higher GDM incidence (RR 2.14, 1.24–3.68), specifically in women with lower mutant-allele burden, and the genotype–intake interaction reached significance (*p* = 0.039). Genetic and dietary determinants of magnesium status would, on this pattern, act upstream of glucose tolerance in pregnancy, with insulin resistance occupying a later position in the sequence, although the interaction reached only nominal significance (*p* = 0.039) and should be read against the multiple comparisons implicit in three SNPs combined with dietary stratification, and against the absence of independent replication.

## 9. Biomarker Methodology: Total Serum Magnesium, Ionized Magnesium and the Magnesium Depletion Score

Nearly every association reviewed so far rests on the same exposure metric of total serum magnesium. The limitations of this metric are well-recognized, given that only about 1% of body magnesium circulates in serum at all, and within that 1% the ionized fraction (roughly 62%) is the only one that is biologically active, the rest being protein-bound (33%) or complexed with small anions (5%) [6,8]. Chronic latent deficiency, in which intracellular and bone stores are already depleted while serum values remain inside the laboratory reference interval, escapes the standard assay [8]. It has been described in Western populations [8], and the cohort literature reviewed here consequently rests on an exposure variable that systematically misses part of its target.

Direct evidence of the scale of that mismatch comes from Al-Maqbali and colleagues, who measured ionized and total magnesium in parallel in 329 adults with T2DM attending a tertiary-care diabetes clinic [54]. Total serum magnesium and ionized magnesium classified hypomagnesemia in 56.2% and 10.0% of the cohort, respectively, with a Spearman correlation of 0.589 and modest agreement on deficiency status. Discordance arose mostly from patients with low total but normal ionized magnesium, with a smaller fraction showing normal total magnesium alongside a low ionized concentration. The two assays capture partly different aspects of circulating magnesium status, and neither has been validated against a reference measure of body magnesium stores, so the direction of error in any individual patient cannot be assigned from the discordance alone. Hypermagnesemia followed the same logic in reverse, with prevalence by ionized assay (11.85%) exceeding that by total magnesium (1.82%). Misclassification therefore operates in both directions, with patients labeled deficient by the routine assay whose ionized concentration sits within the reference range, and patients within the reference range on total magnesium whose ionized concentration is low. Whether this misclassification is non-differential with respect to outcome cannot be established from available data. Hypoalbuminemia, acid-base disturbances and the drugs that alter the protein-bound fraction are themselves associated with cardiovascular and renal risk [6,8], so the error in total magnesium may well be correlated with outcome, and a differential error can bias associations away from the null as readily as toward it. The same measurement caveat applies to nearly all cohort hazard ratios cited in this review and to the parabolic dose–response curve, since these studies used total serum magnesium almost uniformly as their exposure variable; the published estimates remain interpretable as associations with the measured quantity, while their relation to true body magnesium status is uncertain in both magnitude and direction. Internal consistency of those associations across populations and endpoints reduces the likelihood of a purely random association, although shared confounding, publication bias and shared methodological limitations across the field cannot be excluded as partial drivers. The magnitude, optimal cut-off and dose–response shape remain provisional until parallel measurements with ionized magnesium, MDS or 24 h urinary magnesium enter large prospective datasets.

The magnesium depletion score (MDS) addresses these measurement limitations indirectly by integrating four clinical determinants of renal magnesium handling into a composite risk index instead of measuring magnesium at all; it has been associated with clinical event endpoints in several NHANES analyses, although it has not been validated for use in routine clinical practice and does not measure body magnesium stores. Zhang and colleagues took 5219 NHANES 2003–2018 participants with T2DM, stratified MDS into lower (0 to 1 point), middle (2 points) and higher (3 points or more) groups, and showed significant associations of higher MDS with all-cause and cardiovascular mortality in weighted multivariable Cox models, with the effect modified by age above and below 60 years [55]. Ye and colleagues extended the analysis to patients with established cardiovascular disease [56]. Zhou and Yao applied the score to diabetic kidney disease in 3199 NHANES DKD participants, where adjusted hazard ratios for MDS ≥ 3 versus MDS = 0 were 1.78 (95% CI 1.29–2.46) for all-cause mortality and 1.76 (0.94–3.28) for cardiovascular mortality over a median follow-up of 87 months; point estimates for the MDS–mortality association were larger in the subgroups with HbA1c ≥ 7% and with urinary albumin-to-creatinine ratio above 300 mg/g, although formal interaction tests across these strata did not reach statistical significance [57]. Because the individual MDS components may influence outcomes through magnesium-independent pathways, associations between MDS and mortality should not be read as direct evidence that magnesium depletion itself mediates the observed risk. For research purposes, MDS may complement ionized magnesium or 24 h urinary magnesium as a risk index, on the understanding that it indexes exposure to magnesium-lowering conditions and does not measure magnesium status; for routine clinical practice, total serum magnesium remains the default because it is the assay routinely available in clinical laboratories, and is likely to remain so until the alternatives become as accessible and as standardized. None of the proposed alternative biomarkers has yet emerged as a universally validated reference standard (Table 1).

## 10. Magnesium in Diabetic Kidney Disease

### 10.1. Observational and Prospective Evidence

Early evidence in DKD was cross-sectional: Italian T2DM outpatients in the Corsonello series showed lower serum ionized magnesium in microalbuminuric and proteinuric patients than in normoalbuminuric patients [59], and the Pham retrospective series extended the observation to glomerular filtration rate decline [60,61]. The Osaka cohort of Sakaguchi and colleagues delivered the first prospective renal endpoint in T2DM. Among 144 patients with T2DM nephropathy followed for a median of 23 months, the low-magnesium group carried a 2.12-fold higher risk of end-stage kidney disease (95% CI 1.28–3.51, *p* = 0.004) after adjustment for demographic and laboratory covariates. The parallel non-diabetic CKD subgroup of 311 patients followed for 44 months returned an adjusted HR of 1.15 (0.70–1.90, *p* = 0.57) [62], a null result despite a larger absolute number of ESKD events (135 vs. 102) and longer follow-up. Yanagawa and colleagues subsequently reproduced the progression signal in an independent Japanese cohort [63]. Serum magnesium had by then shown prognostic independence in two independent Japanese T2DM cohorts, and the null result in the parallel non-diabetic CKD subgroup raised the possibility that the association is stronger in the diabetic setting. A nonsignificant estimate in one subgroup alongside a significant estimate in another does not establish that the two differ, and no formal test of interaction by diabetes was reported, so evidence of effect modification is lacking (Table 2).

In the Health, Aging and Body Composition study of 2682 older community-dwelling adults followed for ten years, a general-population cohort that did not select on diabetes, dietary magnesium in the upper distribution was independently associated with reduced risk of 30% eGFR decline (IRR per standard deviation 0.79, 95% CI 0.66–0.93) and incident CKD (IRR 0.84, 0.73–0.96), but not with incident cardiovascular disease (HR 0.98, 0.85–1.13) [65]. The renal–cardiovascular split runs against the MR pattern, in which the coronary signal is suggestive and the T2DM signal null, and is consistent with a tubular locus for the renal effect; the dietary-intake design cannot test that mechanism directly, and replication in diabetic populations is needed. Among adults with diabetes, dietary magnesium intake has been linked to mortality in two large cohorts analyzed in parallel by Chi and colleagues: in 6004 NHANES participants with diabetes, the highest versus the lowest intake quartile was associated with lower all-cause, cardiovascular and kidney-disease mortality, the last estimate carrying a wide confidence interval, and in 10,130 UK Biobank participants with diabetes, with lower all-cause and cardiovascular mortality [66]. Dietary intake estimates carry the confounding by overall diet quality discussed in Section 3, and these analyses address mortality, not progression.

Further studies address the drug-exposure and biomarker dimensions of the association. Shah, Sparks and Lee surveyed the SGLT2 inhibitor–magnesium interface in CKD and DKD [67]; a companion case series by Shah, Robbins and Sparks reported two patients with refractory hypomagnesemia whose serum magnesium normalized after SGLT2 inhibitor introduction, without evidence of urinary wasting as the mediating mechanism [68]. Xu, Steele and Fenves described metformin-associated refractory hypomagnesemia in a single case and reviewed the supporting evidence base [69]; Al Maqrashi and colleagues meta-analyzed 23 randomized trials of magnesium supplementation in type 2 diabetes [70]; Xing, Gong and colleagues applied MDS to 3179 NHANES DKD participants and documented graded associations with all-cause and cardiovascular mortality, with adjusted hazard ratios of 1.93 (95% CI 1.34–2.79) and 3.69 (1.70–8.58) respectively in the highest MDS stratum relative to the lowest. Heavy alcohol consumption was the strongest single predictor among the four MDS components, and a separate mediation analysis using metabolic indices identified the estimated glucose disposal rate as a partial mediator accounting for roughly one quarter of the MDS–mortality association; the remaining indices behaved as suppressors in the same models, with cumulative effect insufficient to abolish the underlying signal [64]. The DKD evidence base spans observational cohorts, biomarker analyses and registry data, and two small randomized trials have tested supplementation against albuminuria as a surrogate. Sadeghian and colleagues randomized 80 hypomagnesemic T2DM patients with early nephropathy to magnesium oxide 250 mg daily or placebo for 12 weeks: serum magnesium did not rise significantly, glycemic indices were unchanged, insulin resistance was higher in the magnesium arm at the end of treatment and the lipid profile improved [71]. Halawa and colleagues, in an open-label trial of 60 T2DM patients with CKD stages 3 to 4 and moderately increased albuminuria, reported a reduction in urinary albumin-to-creatinine ratio after 12 weeks of magnesium citrate 2.25 g daily added to standard care, with mild diarrhea the only adverse effect recorded [72]. Neither trial measured kidney failure or sustained eGFR decline, and no randomized trial with such an endpoint appears to have been published. Magnesium status is therefore a prognostic marker in DKD; measurement and, where a deficit is documented, correction within the reference range may be considered, while magnesium remains an untested therapeutic target for the prevention of renal progression.

### 10.2. Prognosis Across the Renal Spectrum

The general-CKD and dialysis literature summarized in this section lies outside the T2DM/DKD framework of the review; it is included, and kept brief, because it defines the shape of the magnesium–mortality relationship at low filtration and the hypermagnesemia risk that constrains dosing in advanced DKD. The downstream end of the renal spectrum is anchored by the Japanese Society for Dialysis Therapy registry analysis of Sakaguchi and colleagues: 142,555 prevalent hemodialysis patients with one-year follow-up and 11,454 deaths, of which 4774 were cardiovascular [73]. Baseline serum magnesium was categorized into sextiles and entered into fully adjusted models. A J-shaped association with all-cause mortality emerged, with the steepest risk in the lowest sextile and the nadir in the fifth (serum magnesium 2.7–3.0 mg/dL). The cardiovascular mortality risk in the highest sextile was attenuated by exclusion of patients with intact parathyroid hormone below 50 pg/mL, locating part of the upper-tail signal within a mineral-bone-disorder phenotype, with a correspondingly smaller share attributable to magnesium itself. The parabolic MACE curve reported by Yin and colleagues in their 1,284,940-patient US veterans T2DM cohort [7] traces a structurally similar pattern, although the curves are not identical: Sakaguchi describes a J, with the steepest excess risk at the low end and a modest uptick at the high end; Yin reports a parabolic curve in which the hazard rises to a higher peak at the upper than at the lower extreme (HRs 1.04–1.39 at high serum magnesium versus 1.11–1.20 at low). Both curves share elevated mortality outside a mid-range serum magnesium window, across two clinically distinct populations.

The mortality observation extends to non-dialysis CKD: in 10,568 Cleveland Clinic patients with eGFR 15–59 mL/min/1.73 m^2^ (Azem and colleagues), three serum-magnesium bands (<1.7, 1.7–2.6, >2.6 mg/dL) were followed over a median of 3.7 years, with 4656 patients (44%) dying during follow-up [74]. Hypomagnesemia carried adjusted HR of 1.14 (95% CI 1.04–1.24) for all-cause mortality and 1.29 (1.12–1.49) for non-cardiovascular non-malignancy death; the upper tail returned HR of 1.23 (1.03–1.48) for all-cause mortality. The U-shape resembles the Sakaguchi dialysis curve and the Yin T2DM parabola in preserved-filtration disease. The eGFR slope did not differ across magnesium groups (*p* = 0.10), implying that the mortality and progression signals operate on partly separate axes.

The general-population evidence complicates any strong claim of diabetes-restricted prognostic value. In 13,226 Atherosclerosis Risk in Communities participants with baseline eGFR at or above 60 mL/min/1.73 m^2^ and a median follow-up of 21 years, low serum magnesium at or below 0.7 mmol/L predicted incident CKD and ESKD with adjusted hazard ratios of 1.58 (95% CI 1.35–1.87) and 2.39 (1.61–3.56), and the associations persisted after exclusion of diuretic users and across strata defined by diabetes and hypertension [75]. The PREVEND cohort reproduced the incident-CKD signal for low plasma magnesium in the general population [76]. These findings locate a magnesium–renal association upstream of established kidney disease and outside the diabetic setting, a pattern compatible with the diabetes-restricted progression signal, since the Sakaguchi comparator addressed progression within established non-diabetic CKD while the ARIC and PREVEND analyses addressed incident disease arising from preserved filtration, so the two need not coincide. In established CKD, the Chronic Renal Insufficiency Cohort extended the mortality analysis to 3867 participants followed for a mean of 14.6 years, where serum magnesium related to all-cause mortality along a nonlinear curve [77], echoing the U-shaped patterns of the Cleveland Clinic and dialysis-registry data.

Phosphate and magnesium appear to operate together: in a non-diabetic predialysis CKD cohort, the Sakaguchi group showed the ESKD risk attributable to higher serum phosphate concentrating in concomitantly hypomagnesemic patients, and attenuating where magnesium levels were normal [78]. Magnesium inhibits calcium–phosphate crystal nucleation and growth in vitro, and if the same inhibition operates in the proximal tubular lumen, magnesium deficit exposes the tubulointerstitium to crystal-induced injury that adequate magnesium would otherwise prevent. The two ions consequently appear to interact as therapeutic targets in CKD, with each modifying the harm attributable to the other, an inference that rests on a single cohort and on in vitro crystallization data. Vascular calcification may track more aggressively in DKD than in non-diabetic CKD of comparable filtration, although head-to-head comparisons remain limited; no direct test in human tissue has addressed the greater weight that magnesium sufficiency might carry in the diabetic setting.

### 10.3. Therapeutic Implications

The single randomized CKD trial of oral magnesium against a vascular imaging endpoint comes from the same Sakaguchi group. This cohort of 125 patients with stage 3–4 CKD (eGFR 15–59 mL/min/1.73 m^2^) and coronary artery calcification (CAC) risk factors, was randomized in a 2 × 2 factorial design to oral magnesium oxide (starting at 330 mg/day, titrated to a serum magnesium target of 2.5–3.0 mg/dL) versus usual care and to oral carbon adsorbent AST-120 versus usual care, with percentage change in coronary artery calcification score by computed tomography as the primary endpoint [79]. Interim analysis stopped the trial early on the magnesium signal: median CAC change was 11.3% in the magnesium oxide arm against 39.5% in controls, with rapid progressors (annualized CAC change ≥15%) running at 23.9% versus 62.0%; AST-120 had no effect on CAC progression. Magnesium oxide did not suppress thoracic aorta calcification, and 27% of patients in the magnesium oxide arm dropped out against 17% of controls, mainly because of diarrhea. Sample size and population breadth both limit the trial, and coronary artery calcification progression stands as an imaging surrogate distinct from an adjudicated cardiovascular event, so the result supports an effect on calcification while leaving clinical outcomes untested; it remains the only randomized evidence in CKD against a vascular imaging endpoint.

Two levels of clinical guidance should be distinguished. Current KDIGO guidance recommends SGLT2 inhibitors and, in eligible patients with T2DM and CKD, GLP-1 receptor agonists on cardiovascular and renal grounds, and addresses medication review in people with reduced filtration [2]; neither KDIGO nor ADA guidance addresses magnesium screening or repletion. The considerations that follow are therefore hypothesis-based and derived from the observational literature reviewed above, and none carries guideline standing. Serum magnesium measurement is inexpensive and widely available in hospital laboratories, and uptake of repletion among biochemically eligible outpatients is low [7]. Where hypomagnesemia is documented and a magnesium-wasting co-medication carries a discretionary indication, review of that exposure before supplementation is consistent with general principles of medication rationalization in CKD; proton-pump inhibitors are the most amenable where clinical indication permits, and thiazide and loop diuretics can be reviewed for dose where tolerated. Metformin is a different case, since its cardiorenal benefit weighs heavily against discontinuation and the relevant decision concerns the addition of repletion. SGLT2 inhibitor therapy, indicated on nephroprotective grounds independent of magnesium status, may shift serum magnesium toward the middle of the reference interval as a secondary effect. Where deficiency persists after upstream adjustments, oral magnesium repletion may be considered, with dose adjusted for filtration rate and serum magnesium reassessed at an interval appropriate to the clinical context. Empirical supplementation in normomagnesemic DKD patients, and substitution of magnesium repletion for established nephroprotective interventions, have no support in current evidence.

The SGLT2 inhibitor effect on serum magnesium requires separate consideration in the DKD setting. The 18-trial meta-analysis of Tang and colleagues [80] and the TriNetX target trial emulation of Shao and colleagues [81] are both anchored in the broader T2DM population, and the trials contributing to the pooled SGLT2i–magnesium estimates typically excluded patients with eGFR below 30 mL/min/1.73 m^2^ and enrolled sparsely in the 30–60 range. Subgroup analyses in Shao are aligned in direction with the main-effect estimate, showing lower hypomagnesemia risk across the eGFR 30–60 and eGFR ≥ 60 strata and across patients with or without a CKD diagnosis, although granularity remains lacking at the intersections that matter most in DKD, in particular advanced disease (eGFR 15–30) and concurrent mineral-bone disorder. The DAPA-CKD and EMPA-KIDNEY populations, which include substantial numbers of DKD patients with reduced filtration and meaningful albuminuria, have not been reanalyzed for magnesium-specific outcomes to date. No data establish that the modest SGLT2i-induced shift of serum magnesium toward the middle of the reference interval is preserved in advanced DKD, and the question carries direct bearing on safety monitoring and on the interpretation of the nephroprotective benefit observed in those trials.

The hypermagnesemia risk at low filtration rate sits in its own right, separate from the deficiency signal that dominates the literature. The Azem analysis returned a hazard ratio of 1.23 for all-cause mortality at the upper tail in an eGFR 15–59 mL/min/1.73 m^2^ population [74], and the Sakaguchi 2014 registry reproduced the excess-risk signal at the high end among prevalent hemodialysis patients [73]; the prognostic penalty at the top of the distribution is consequently not an artifact of end-stage disease. Standard oral doses of 300 to 450 mg elemental magnesium daily, calibrated against trials in patients with preserved renal function, may overshoot in advanced DKD where passive and active renal excretion are both compromised. In the absence of dose-finding data in this population, these considerations support cautious dosing and biochemical monitoring in advanced DKD, with serum magnesium reassessed before further titration; the evidence is insufficient to define a specific dose-reduction strategy or an appropriate target concentration in advanced DKD, and this framing represents one cautious approach and not a guideline-derived recommendation.

## 11. Randomized Controlled Trials of Magnesium Supplementation

The interventional evidence base is smaller than the observational one, and the earliest adequately controlled trial, by Rodríguez-Morán and Guerrero-Romero, randomized 63 hypomagnesemic T2DM patients to 50 mL daily of an oral magnesium chloride solution (50 g MgCl_2_ per liter, delivering approximately 450 mg of elemental magnesium daily) or placebo for sixteen weeks, with significant improvements in fasting glucose, HbA1c and HOMA-IR in the magnesium arm [27]. A follow-on study by Guerrero-Romero and Rodríguez-Morán moved the question to non-diabetic hypomagnesemic adults, gave oral magnesium chloride at 382 mg elemental magnesium per day for three months, and recorded a rise in the disposition index, consistent with improved beta-cell compensation for a given level of peripheral insulin sensitivity [28].

Two recent double-blind trials extend this evidence in opposite directions. Drenthen and colleagues, in a placebo-controlled crossover study of 14 insulin-treated T2DM patients with serum magnesium at or below 0.79 mmol/L, gave magnesium gluconate at 15 mmol (360 mg) daily for six weeks: serum magnesium rose by 0.05 mmol/L, whereas the glucose infusion rate during hyperinsulinemic-euglycemic clamp, the primary endpoint, did not differ from placebo, and HbA1c, continuous glucose monitoring metrics, insulin dose and blood pressure were also unchanged [29]. Al-Maqbali and colleagues randomized 247 T2DM patients with HbA1c of 7% or higher, of whom fewer than a tenth were hypomagnesemic at baseline, to magnesium oxide 500 mg (302 mg elemental) or placebo for 12 months: the primary endpoint, change in HbA1c, did not differ significantly between arms, fasting glucose fell in the magnesium arm in the subset with available data, and the HbA1c reduction was numerically larger among participants with baseline hypomagnesemia, a subgroup of 18 patients in which the difference did not reach significance [82]. The two trials differ in formulation, dose, duration, baseline magnesium status and endpoint, and together they indicate that a modest rise in serum magnesium in patients whose values already sit close to the reference range produces little measurable change in insulin sensitivity or glycemic control.

Multiple meta-analyses have addressed the supplementation evidence: Simental-Mendía and colleagues pooled randomized trials of magnesium supplementation on insulin sensitivity and glucose control and reported a significant reduction in HOMA-IR (WMD −0.67, 95% CI −1.20 to −0.14, *p* = 0.013) without a significant primary-analysis effect on fasting plasma glucose, HbA1c or insulin; in the subgroup with supplementation lasting four months or longer, both fasting glucose and HOMA-IR improved significantly [83]. Verma and Garg extended the analysis to cardiovascular risk factors in T2DM, with significant improvements in fasting plasma glucose, lipid profile and systolic blood pressure, and a larger effect in the hypomagnesemic subgroup [84]. Asbaghi and colleagues, restricting to blood pressure and anthropometric outcomes, returned congruent reductions in systolic and diastolic pressures [85]. Veronese and colleagues pooled double-blind trials in people with or at risk of diabetes [86]. Magnesium supplementation lowered fasting plasma glucose in the diabetic stratum and improved fasting plasma glucose, 2 h oral glucose tolerance and (in meta-regression) HOMA-IR in the at-risk-of-diabetes stratum; HbA1c and fasting insulin signals were null. The An meta-analysis in JACC, with 884 randomized trials and 883,627 participants, placed magnesium among the micronutrients with moderate-to-high-quality evidence for reducing cardiovascular disease risk factors; reductions in adjudicated cardiovascular events in that dataset were most clearly attributable to n-3 fatty acids [87]. The trials underlying these syntheses differ in formulation (magnesium chloride solution, oxide, citrate, gluconate and chelated salts), in elemental dose (roughly 250 to 450 mg daily), in duration (six weeks to twelve months), in the baseline status of participants (selected for hypomagnesemia in the Durango and Nijmegen trials, unselected in most others) and in endpoints, which are almost uniformly glycemic and cardiometabolic intermediates (Table 3). Within this heterogeneity, effect direction is reproducible across syntheses, effect magnitude is larger where baseline deficiency was documented and supplementation lasted four months or longer [83,84], and no trial has been powered for clinical events; the evidence supports targeted correction of a documented deficit and gives no basis for indiscriminate supplementation.

Across these syntheses, the direction of effect is consistent with baseline status: supplementation improves glycemic and cardiovascular intermediates primarily in patients with documented deficiency. The insulin–TRPM6 framework would accommodate this pattern if repletion interrupted the pathway only where magnesium is rate-limiting, although the clamp study of Drenthen and colleagues shows that a rise in serum magnesium does not by itself restore insulin sensitivity [29]. Yin and colleagues reproduced the same pattern at event level in a new-user-design propensity-score matched cohort analysis, where oral magnesium was associated with lower MACE in hypomagnesemic T2DM and with a null result in normomagnesemic T2DM [7]; the analysis is observational and offers support short of confirmation. Randomized data therefore show modest effects on glycemic intermediates that concentrate in deficient patients, and observational data show lower event rates under repletion in the same group; the randomized trials have not measured clinical events and the observational analysis cannot establish causation, so the case for targeted correction rests on the convergence of two incomplete lines of evidence.

## 12. Drug Interactions

T2DM pharmacotherapy exerts effects on serum magnesium, with SGLT2 inhibitors raising it while thiazide and loop diuretics and proton-pump inhibitors lower it through gastrointestinal and renal pathways; metformin has been linked to lower levels less consistently. GLP-1 receptor agonists shift it upward less than SGLT2 inhibitors [81]. Effects are individually modest, although concurrent exposures may produce clinically relevant deficits, with implications for both interpretation and management in the individual T2DM patient.

The SGLT2 inhibitor signal is reproducible across randomized syntheses and small in magnitude. In 18 randomized trials of 15,309 T2DM patients across four SGLT2 inhibitors, the Tang meta-analysis returned placebo-adjusted weighted mean differences in serum magnesium between 0.04 and 0.10 mmol/L, with within-agent dose dependence (canagliflozin 100/300 mg: 0.06 and 0.09; empagliflozin 10/25 mg: 0.04 and 0.07) and the largest single-dose effect at dapagliflozin 10 mg (0.10), against ipragliflozin 50 mg (0.05) [80]. Translation to hazard terms came from the Shao TriNetX target trial emulation, with 1:1 propensity-score matching across three contrasts: SGLT2 inhibitor against DPP-4 inhibitor (n = 718,798), GLP-1 receptor agonist against DPP-4 inhibitor (n = 623,390), and SGLT2 inhibitor against GLP-1 receptor agonist (n = 702,808) [81]. Incident hypomagnesemia was lowest under SGLT2 exposure (HR 0.80, 95% CI 0.79–0.82, against DPP-4), intermediate under GLP-1 agonists (HR 0.89, 0.88–0.91, against DPP-4), with the SGLT2-versus-GLP-1 head-to-head favoring SGLT2 (HR 0.92, 0.91–0.93); within-class effects did not differ materially. Patient-level analyses resolved the dependence on baseline status, as shown in the canagliflozin pooled post hoc analysis of Gilbert and colleagues, in which 74.1% of hypomagnesemic patients on canagliflozin 100 mg and 80.6% on canagliflozin 300 mg reached the reference interval (≥0.74 mmol/L) at week 26, against 28.8% on placebo [88]. The parallel pooled analysis of Toto and colleagues across ten dapagliflozin trials returned a 47.8 percentage-point higher proportion of hypomagnesemic patients reaching the reference interval under active treatment versus placebo (95% CI 41.4–53.9), with a parallel within-class shift in serum magnesium concentrated among baseline-hypomagnesemic patients and minimal movement among normomagnesemic ones [89]. Three patients with refractory diabetic hypomagnesemia, in whom canagliflozin, empagliflozin or dapagliflozin restored normomagnesemia (one discontinuing daily intravenous replacement, two with reduction in fractional magnesium excretion), illustrate the same mechanism in the extreme phenotype [90].

The mechanism remains incompletely characterized [67], and proposed contributors include altered solute delivery to the thick ascending limb and the distal convoluted tubule, glycemic-control-dependent restoration of insulin-mediated TRPM6 trafficking, and direct or indirect effects on TRPM6 expression and on paracellular permeability via claudin-16/19 [5,67,90]. Human tubular confirmation for any of these candidate mechanisms remains limited. SGLT2 inhibition shifts patients away from the lower end of the serum magnesium distribution toward mid-range. A magnesium-related component of the cardiovascular and renal benefit observed in the SGLT2 inhibitor outcome trials remains a hypothesis at present, untested against the trial data, since no formal mediation analysis within those datasets appears to have been published.

Metformin has been associated with lower serum magnesium, although the evidence is inconsistent, with intestinal handling the most discussed locus of the proposed effect. The Fremantle Diabetes Study linked metformin exposure to lower serum magnesium and, within the hypomagnesemic subgroup, to higher cardiovascular risk [91]. Proposed mechanisms include altered intestinal magnesium handling and bile-acid-dependent absorption pathways [69]. However, the dominant contributor in vivo has not been established. Metformin-treated hypomagnesemic patients would appear a logical repletion target, although in the Yin propensity-score matched analysis the MACE benefit of oral magnesium did not extend to the metformin-exposed subgroup, a null that may reflect genuine drug-context specificity or the power limits of subgroup analysis.

Thiazide and loop diuretics are the most thoroughly characterized pharmacological causes of hypomagnesemia. Loop diuretics inhibit paracellular magnesium reabsorption at the thick ascending limb by abolishing the lumen-positive transepithelial gradient; thiazides downregulate TRPM6 expression at the distal convoluted tubule. Hypertensive T2DM patients are commonly exposed to one or the other, sometimes both, and baseline hypomagnesemia prevalence tracks diuretic burden.

Proton-pump inhibitors drive hypomagnesemia through the intestine, although the dominant in vivo contributor is not fully established. Proposed mechanisms include reduced solubilization of magnesium salts at higher gastric pH and indirect attenuation of TRPM6-mediated colonic absorption. Pooled estimates from nine observational studies covering 109,798 participants, drawn from general and hospital populations and not restricted to diabetes, placed the relative risk at 1.43 (95% CI 1.08–1.88), rising to 1.63 (1.14–2.23) when restricted to higher-quality studies [92]. The Yin veterans propensity-score matched analysis adds an interaction term: within the hypomagnesemic cohort, the lower-MACE estimate under prescribed oral magnesium was modified by baseline proton-pump inhibitor use (*p* = 0.026 for interaction) and by thiazide use (*p* = 0.039), with a larger estimated benefit in patients exposed to these drugs [7]. Drug-induced deficits consequently define the context in which the repletion signal is largest, although whether this reflects genuine drug-context-specific rescue or selection of patients more likely to benefit cannot be resolved from observational data. Deprescription, where clinically feasible, would be the upstream complement to supplementation, on the hypothesis-based footing described in Section 10.3.

GLP-1 receptor agonists act in the same direction as SGLT2 inhibitors on magnesium balance but with a smaller effect size. In the TriNetX target trial emulation, GLP-1 agonist initiation was associated with lower incident hypomagnesemia relative to DPP-4 inhibitors, and was itself outperformed by SGLT2 inhibitors in the head-to-head comparison [81]. Mechanistic accounts remain speculative, since improvements in insulin sensitivity would restore TRPM6 function through the same insulin-dependent pathway, while direct effects on gut–kidney signaling have been proposed and await demonstration in human tissue.

Concurrent exposure to multiple depleting drugs defines the practical risk: a patient receiving metformin alongside a thiazide and a proton-pump inhibitor carries three depleting drugs on top of the insulin-resistance substrate; SGLT2 inhibitor introduction reduces but does not abolish the cumulative deficit. Periodic serum magnesium monitoring is inexpensive, and the Yin veterans data document substantial undertreatment, with prescribed oral magnesium reaching only 9.7% of hypomagnesemic patients [7]. Routine substitution of ionized magnesium or MDS for total serum magnesium has no guideline backing at present, and uptake will depend on assay availability and on validation in large prospective datasets.

### Calcineurin Inhibitors and Post-Transplant Diabetes Mellitus

Post-transplant diabetes is included because the transplant setting supplies a temporal sequence—drug exposure followed by hypomagnesemia followed by incident diabetes—that the cross-sectional T2DM literature cannot provide; the section is confined to that point. Calcineurin inhibitors, given to prevent allograft rejection, are a recognized iatrogenic cause of hypomagnesemia, more pronounced with tacrolimus than with ciclosporin. The deficit originates in renal magnesium wasting: tacrolimus binds FKBP12 to inhibit calcineurin [93] and downregulates TRPM6 at the distal convoluted tubule, raising urinary magnesium loss [94].

Navaneethan and colleagues quantified the renal component in 41 transplant recipients against 10 healthy volunteers, with 43% of the tacrolimus group hypomagnesemic and fractional excretion of magnesium reaching 7.42 ± 3.59% against 1.88 ± 0.43% in controls, and 24 h urinary magnesium correspondingly higher [95].

Transplant recipients offer an analytic advantage that the cross-sectional T2DM literature cannot provide, since the magnesium-wasting drug is introduced at a known time, the serum deficit follows, and incident diabetes can be observed prospectively in that order. Hypomagnesemia during immunosuppression is consequently a recognized antecedent of post-transplant diabetes mellitus (PTDM). In the retrospective cohort of Van Laecke and colleagues, each 1 ng/mL increase in blood tacrolimus concentration carried a 33% higher adjusted risk of PTDM (HR 1.332, 95% CI 1.039–1.707, *p* = 0.024), while each 0.1 mg/dL increment in serum magnesium carried a 31% lower risk (HR 0.691, 95% CI 0.569–0.839, *p* < 0.001), magnesium entering the model as an independent predictor [96].

The prospective series of van der Burgh and colleagues reproduced the antecedent relationship: 29 of 167 kidney recipients (17%) developed PTDM within the first year, higher tacrolimus concentrations predicted incident hypomagnesemia (OR 1.28, 95% CI 1.09–1.50, *p* = 0.003), and patients who progressed to PTDM followed a lower serum magnesium trajectory throughout [97].

## 13. Gestational Diabetes Mellitus

Pregnancy differs from the non-pregnant T2DM setting: gestational insulin resistance arises from placental hormones over a matter of weeks, where the T2DM phenotype accumulates over years through adiposity and physical inactivity. Maternal magnesium requirement increases in pregnancy on account of expanded plasma volume and fetal–placental uptake, and the recommended dietary allowance is raised accordingly [58]; mild hypomagnesemia is common even in uncomplicated gestation and more frequent still in gestational diabetes. Gestational diabetes is included here because it offers a model in which insulin resistance develops over weeks and because the gene–environment analysis discussed below is one of few datasets to place magnesium handling upstream of glucose tolerance. The maternal metabolic environment in gestational diabetes affects fetal growth and neonatal outcomes through pathways reviewed elsewhere [98], and maternal nutritional status interacts with microbiome-derived metabolites, oxidative stress and epigenetic regulation in shaping perinatal outcomes [99]; magnesium is one element within that broader nutritional context.

Observational meta-analyses report lower serum magnesium in GDM than in normoglycemic pregnancy: Ren and colleagues pooled 17 studies with 2858 participants and found the difference most pronounced in the third trimester [100], and a Turkish case–control series reported lower ionized and total magnesium in women with gestational diabetes than in normoglycemic pregnant controls [101]. Interventional data are limited to small trials pooled in three overlapping meta-analyses, which report reductions in fasting plasma glucose [102,103] and, in the largest, in HOMA-IR, without detectable effects on serum insulin, preterm delivery or macrosomia [104]; between-study heterogeneity was lower than for most other micronutrient–GDM associations, and pooled magnitudes are not reproduced here given the size of the contributing trials.

The TRPM6/TRPM7 gene–environment interaction analysis by Cao and colleagues contributes the most distinctive piece of GDM–magnesium evidence published to date [53]. The asymmetric structure of the interaction is the principal finding: magnesium intake below 370 mg/day raises GDM risk specifically in women with fewer TRPM6/TRPM7 mutant alleles, whereas women with a higher mutant-allele burden sit at elevated baseline risk regardless of intake. One interpretation places magnesium handling upstream of glucose tolerance in pregnancy, inverting the order that appears more likely in non-pregnant T2DM, where circulating magnesium behaves in part as a downstream readout of insulin resistance; the evidence comes from a single cohort and requires replication before any causal direction can be considered established. If confirmed, the practical implication could extend to attention to dietary magnesium during pregnancy, with additional consideration in populations where TRPM6/TRPM7 variants are common. A trial designed to test this hypothesis would extend the single-cohort evidence, and none appears to have been reported.

## 14. Research Priorities

A randomized controlled trial of magnesium supplementation with a DKD progression endpoint would address the principal gap in the DKD evidence: baseline magnesium status characterized by ionized magnesium, with MDS recorded as a complementary risk index, stratification by insulin-resistance indices, and a composite kidney endpoint aligned with contemporary trials, such as sustained decline in eGFR of at least 40%, kidney failure or death from renal causes. Direct comparison, cross-sectional and longitudinal, of serum magnesium, ionized magnesium and MDS in DKD versus non-diabetic CKD patients matched for filtration rate, albuminuria and medication exposure would move the DKD-restricted pattern from circumstantial to tested. Mechanistic work on SGLT2 inhibitor effects on TRPM6 expression and on paracellular magnesium reabsorption in the thick ascending limb would identify the dominant pathway underlying the clinical serum magnesium response and clarify whether combination strategies are additive. A head-to-head target trial emulation comparing SGLT2 inhibitor, GLP-1 receptor agonist and combination therapy on serum magnesium trajectory in T2DM, with ionized magnesium or MDS in place of total serum magnesium as the exposure variable, would extend the comparative evidence base beyond what TriNetX has so far produced. Preventive trials of dietary magnesium optimization in pregnancy, with women carrying TRPM6/TRPM7 variants identified as the higher-risk stratum, remain to be undertaken to test directly whether magnesium handling acts as an upstream determinant of glucose tolerance during gestation. Formal dose-finding and safety data for oral magnesium repletion in CKD stages 4 and 5 are absent in a population where renal magnesium excretion is already compromised; standard doses calibrated against preserved-function cohorts risk overshooting into the prognostically unfavorable upper tail of the serum distribution, and current dosing recommendations rest on extrapolation, with no dedicated evidence in CKD stages 4 and 5.

## 15. Conclusions

Hypomagnesemia is common in T2DM and more common in DKD, and the evidence reviewed here is compatible with insulin-resistance physiology acting as one upstream determinant of renal magnesium wasting alongside dietary intake and drug exposure; the data do not discriminate between magnesium as a causal mediator, a modifier or a biomarker of insulin-resistant physiology, and the mechanism rests largely on cellular and experimental work. In observational cohorts, low serum magnesium is associated with poorer glycemic control, with cardiovascular and microvascular events and, in DKD, with progression to kidney failure, with the measurement caveats of total serum magnesium applying throughout. Randomized trials show modest effects on glycemic intermediates that concentrate in patients with documented deficiency, and clinical events have not been tested; a propensity-matched analysis of prescribed repletion in hypomagnesemic veterans adds an observational layer that cannot substitute for a trial. On this evidence, magnesium status is a reproducible prognostic marker, measurement may be considered in T2DM patients exposed to magnesium-wasting drugs and in DKD, and targeted correction of a documented deficit is a reasonable clinical consideration; empirical supplementation in normomagnesemic patients has no support, and none of these considerations carries guideline standing. Randomized trials with renal endpoints remain the principal evidence gap.

## Figures and Tables

**Table 1:** Magnesium physiology, recommended intake and oral supplement formulations. (**A**) Serum reference values and body distribution. (**B**) Recommended dietary allowance (US Institute of Medicine) [58]. (**C**) Oral magnesium formulations: elemental content and bioavailability.

**(A)**				
**Parameter**		**Value**		**Source**
Total body Mg content		22–26 g (~1000 mmol)		[6]
Bone		~60% of total		[6]
Skeletal muscle		~20%		[6]
Other soft tissues		~19%		[6]
Extracellular compartment		~1%		[6]
Serum ionized fraction		~62%		[6,8]
Serum protein-bound fraction		~33%		[6,8]
Serum complexed fraction		~5%		[6,8]
Reference interval, total serum Mg		0.75–0.95 mmol/L (1.8–2.3 mg/dL)		[6]
Hypomagnesemia threshold (common)		<0.75 mmol/L (<1.8 mg/dL)		[5,24]
Hypomagnesemia threshold (strict)		<0.70 mmol/L (<1.7 mg/dL)		[19,20]
**(B)**				
**Group**		**Age**		**RDA (mg/d)**
Males		19–30 y		400
Males		31+ y		420
Females		19–30 y		310
Females		31+ y		320
Pregnancy		19–30 y		350
Pregnancy		31–50 y		360
Lactation		19–30 y		310
Lactation		31–50 y		320
UL (supplemental only)		Adults		350
**(C)**				
**Formulation**	**Elemental Mg (%)**	**Comparative Absorption Data**	**GI Tolerability**	**Notes**
Mg oxide	~60%	Low; poorly soluble salt	Laxative effect	Highest elemental content; used in RCTs [27]; poor absorption limits efficacy for repletion
Mg chloride	~12%	Better absorbed than oxide	Generally well tolerated	Used in foundational RCTs [27,28]; good solubility
Mg glycinate	~14%	Comparative human data limited	Well tolerated	Chelated form; reported to be well tolerated in chronic use
Mg lactate	~12%	Comparative human data limited	Well tolerated	Organic salt; used as an alternative to oxide for repletion
Mg taurate	~9%	Comparative human data limited	Well tolerated	Limited clinical data in diabetes; no outcome data
Mg L-threonate	~8%	Comparative human data limited	Well tolerated	Studied for cognitive endpoints on the basis of central nervous system penetration; no data in diabetes or CKD

Threshold values reflect commonly applied cut-offs in published cohort studies; no current diabetes or kidney guideline endorses a single universal threshold. Mg, magnesium; RDA, recommended dietary allowance; UL, tolerable upper intake level (supplemental sources only); GI, gastrointestinal. Comparative absorption entries are qualitative and derive from small pharmacokinetic studies in healthy volunteers; they depend on formulation, dose, gastric pH and individual patient factors, and none has been tested against clinical endpoints in diabetes or CKD. Organic salts (citrate, glycinate, lactate) are generally better absorbed than inorganic salts (oxide, carbonate). Entries in this panel are descriptive and should not be read as comparative clinical recommendations.

**Table 2:** Observational and cohort studies of magnesium in diabetic kidney disease.

Study [Ref]	Design	Population	N	Exposure	Follow-up	Key Findings
Corsonello et al. 2000 [59]	Cross-sectional	T2DM outpatients, Italy	90 (plus 20 healthy controls)	Ionized serum Mg	–	Reduced ionized Mg in microalbuminuric and proteinuric vs. normoalbuminuric T2DM
Pham et al. 2005 [60]	Retrospective	T2DM, Olive View-UCLA	550	Total serum Mg	63 months	Lower Mg associated with steeper 1/SCr slope; independent of HTN, ACEI/ARB, diuretics, statins, aspirin
Pham et al. 2009 [61]	Retrospective (same cohort)	T2DM, Olive View-UCLA	329 (tot.550)	Total serum Mg	97 months	Lowest Mg group: SCr ratio 1.54; 22.2% developed overt proteinuria vs. 0% in highest group
Sakaguchi et al. 2012 [62]	Prospective cohort	T2DM nephropathy, Japan	144	Total serum Mg	23 months	Low-Mg group HR 2.12 (1.28–3.51) for ESKD; null in non-diabetic CKD (n = 311)
Yanagawa et al. 2021 [63]	Retrospective	T2DM on OADs, Japan	690	Total serum Mg	Variable	HypoMg associated with DKD progression per KDIGO risk categories
Zhou and Yao 2025 [57]	NHANES cohort	DKD (NHANES)	3199	MDS	Longitudinal	Graded MDS-mortality; strongest at HbA1c ≥ 7% and ACR > 300 mg/g
Xing, Gong et al. 2025 [64]	NHANES cohort	DKD (NHANES)	3179	MDS	Longitudinal	Graded MDS association with all-cause and cause-specific mortality

T2DM, type 2 diabetes mellitus; DKD, diabetic kidney disease; Mg, magnesium; eGFR, estimated glomerular filtration rate; ESKD, end-stage kidney disease; CKD, chronic kidney disease; HD, hemodialysis; JSDT, Japanese Society for Dialysis Therapy; CV, cardiovascular; OADs, oral antidiabetic drugs; KDIGO, Kidney Disease: Improving Global Outcomes; MDS, magnesium depletion score; NHANES, National Health and Nutrition Examination Survey; HbA1c, glycated hemoglobin; ACR, albumin-to-creatinine ratio; SCr, serum creatinine; HR, hazard ratio; HTN, hypertension; ACEI, angiotensin-converting enzyme inhibitor; ARB, angiotensin receptor blocker.

**Table 3:** Randomized controlled trials, one uncontrolled study and meta-analyses of magnesium supple-mentation in diabetes mellitus and diabetic kidney disease.

Study [Ref]	Design	Population	N	Intervention	Duration	Primary Outcomes
Rodriguez-Moran and Guerrero-Romero 2003 [27]	RCT, double-blind	Hypomagnesemic T2DM	63	MgCl_2_ solution (~450 mg/d elemental Mg) vs. placebo	16 wk	Significant reduction in fasting glucose, HbA1c and HOMA-IR
Guerrero-Romero and Rodriguez-Moran 2011 [28]	RCT, double-blind	Hypomagnesemic non-diabetic adults	106 (97 completed)	MgCl_2_ (382 mg/d elemental Mg) vs. placebo	3 months	Improved disposition index (beta-cell compensation)
Shahbah et al. 2017 [21]	Open-label, single-center, uncontrolled (not randomized)	Hypomagnesemic children, T1DM	20 (of 71 screened)	MgO 300 mg/d	3 mo	Serum Mg rose; HbA1c 10.11% to 7.88% (*p* < 0.001)
Sadeghian et al. 2020 [71]	RCT, double-blind	Hypomagnesemic T2DM with early nephropathy	80	MgO 250 mg/d vs. placebo	12 wk	Serum Mg unchanged; glycemic indices unchanged; insulin resistance higher in Mg arm; lipid profile improved
Halawa et al. 2023 [72]	RCT, open-label	T2DM, CKD stages 3–4, microalbuminuria; low or normal serum Mg	60 (54 completed)	Mg citrate 2.25 g/d plus standard care vs. standard care	12 wk	Reduction in UACR (primary endpoint); mild diarrhea only reported adverse effect
Drenthen et al. 2024 [29]	RCT, double-blind, crossover	Insulin-treated T2DM, serum Mg ≤ 0.79 mmol/L	14	Mg gluconate 15 mmol/d (360 mg) vs. placebo	6 wk per period	Clamp glucose infusion rate unchanged (primary endpoint); HbA1c, CGM metrics, insulin dose and BP unchanged
Al-Maqbali et al. 2026 [82]	RCT, double-blind	T2DM with HbA1c ≥7%; <10% hypomagnesemic at baseline	247	MgO 500 mg/d (302 mg elemental) vs. placebo	12 mo	HbA1c change not significant (primary endpoint); FBG reduced; larger HbA1c reduction in hypomagnesemic subgroup, not significant
Simental-Mendia et al. 2016 [83]	SR + MA of RCTs	T2DM and non-diabetic	Pooled	Oral Mg supplementation	Variable	Reduced HOMA-IR; no significant effect on FPG, HbA1c or insulin in the primary analysis; FPG and HOMA-IR improved in trials lasting ≥4 months
Verma and Garg 2017 [84]	SR + MA of RCTs	T2DM	Pooled	Oral Mg supplementation	Variable	Improved FPG, HDL, LDL, TG and SBP; larger effect in hypoMg subgroups
Asbaghi et al. 2021 [85]	SR + MA of RCTs	T2DM	Pooled	Oral Mg supplementation	Variable	Reduced SBP and DBP
Veronese et al. 2021 [86]	SR + MA of RCTs	T2DM and at risk of diabetes	Pooled	Oral Mg supplementation	Variable	Glucose-insulin improvements; hypoMg-subgroup pattern confirmed
An et al. 2022 [87]	Umbrella MA	General (CV risk)	Pooled	Micronutrient supplementation	Variable	Mg associated with favorable effects on selected cardiometabolic risk factors; hard-outcome evidence less definitive
Al Maqrashi et al. 2025 [70]	SR + MA of 23 RCTs	T2DM	Pooled	Oral Mg supplementation	Variable	Improved glucose, blood pressure and lipid profile

RCT, randomized controlled trial; SR, systematic review; MA, meta-analysis; T2DM, type 2 diabetes mellitus; T1DM, type 1 diabetes mellitus; Mg, magnesium; MgCl_2_, magnesium chloride; MgO, magnesium oxide; HbA1c, glycated hemoglobin; HOMA-IR, homeostatic model assessment of insulin resistance; FPG, fasting plasma glucose; SBP, systolic blood pressure; DBP, diastolic blood pressure; NDS, Neuropathy Disability Score; NS, not significant; CV, cardiovascular; hypoMg, hypomagnesemia; UACR, urinary albumin-to-creatinine ratio; CGM, continuous glucose monitoring; BP, blood pressure. Trials are ordered by year; the references [21,29,71,72,82] entries are single trials, all others are pooled syntheses. The reference [21] was open-label without parallel control; concurrent intensification of routine diabetes care cannot be excluded.

## Data Availability

No new data were created or analyzed in this study. Data sharing is not applicable to this article.

## Abbreviations

The following abbreviations are used in this manuscript:

ACEI	angiotensin-converting enzyme inhibitor
ACR	albumin-to-creatinine ratio
ADA	American Diabetes Association
AGE	advanced glycation end-product
ANOVA	analysis of variance
ARB	angiotensin receptor blocker
ARIC	Atherosclerosis Risk in Communities study
AST-120	oral spherical carbon adsorbent
CAC	coronary artery calcification
CAD	coronary artery disease
CARDIoGRAMplusC4D	Coronary Artery Disease Genome-wide Replication and Meta-analysis plus the Coronary Artery Disease Genetics consortium
CHARGE	Cohorts for Heart and Aging Research in Genomic Epidemiology
CI	confidence interval
CKD	chronic kidney disease
CNI	calcineurin inhibitor
CNS	central nervous system
CV	Cardiovascular
DBP	diastolic blood pressure
DCS	Diabetes Care System (Dutch cohort)
DCT	distal convoluted tubule
DKD	diabetic kidney disease
DPP-4	dipeptidyl peptidase-4
eGFR	estimated glomerular filtration rate
ESKD	end-stage kidney disease
FEMg	fractional excretion of magnesium
FKBP12	FK506-binding protein 12
FPG	fasting plasma glucose
GDM	gestational diabetes mellitus
GI	Gastrointestinal
GLP-1 RA	glucagon-like peptide-1 receptor agonist
GLUT4	glucose transporter type 4
GWAS	genome-wide association study
HbA1c	glycated hemoglobin
HDL	high-density lipoprotein
HNF1β	hepatocyte nuclear factor 1β
HOMA-IR	homeostatic model assessment of insulin resistance
HR	hazard ratio
hsCRP	high-sensitivity C-reactive protein
HTN	Hypertension
IDF	International Diabetes Federation
IRR	incidence rate ratio
IU	international unit
IVW	inverse-variance weighted
JSDT	Japanese Society for Dialysis Therapy
KDIGO	Kidney Disease: Improving Global Outcomes
LDL	low-density lipoprotein
MA	meta-analysis
MACE	major adverse cardiovascular events
MDS	magnesium depletion score
Mg	Magnesium
MgCl_2_	magnesium chloride
MgO	magnesium oxide
MNSI	Michigan Neuropathy Screening Instrument
MR	Mendelian randomization
NHANES	National Health and Nutrition Examination Survey
NKCC2	Na^+^-K^+^-2Cl^−^ cotransporter 2
NODAT	new-onset diabetes after transplantation
OAD	oral antidiabetic drug
OR	odds ratio
PI3K	phosphoinositide 3-kinase
PTDM	post-transplant diabetes mellitus
Rac1	Ras-related C3 botulinum toxin substrate 1
RCT	randomized controlled trial
RDA	recommended dietary allowance
ROC	receiver operating characteristic
ROMK	renal outer medullary potassium channel
SBP	systolic blood pressure
SCr	serum creatinine
SGLT2	sodium-glucose cotransporter 2
SNP	single nucleotide polymorphism
SR	systematic review
T1DM	type 1 diabetes mellitus
T2DM	type 2 diabetes mellitus
TG	Triglycerides
TIRF	total internal reflection fluorescence
TRPM6	transient receptor potential melastatin 6
TRPM7	transient receptor potential melastatin 7
UL	tolerable upper intake level
VA	Veterans Affairs
WHO	World Health Organization
WMD	weighted mean difference
